# Operation for Hare-Lip, with Removal of the Intermaxillary Bone

**Published:** 1872-01

**Authors:** 


*Operation for Hare-lip, with Removal of the Intermaxillary Bone.*—One operation had already been performed on this patient, who was about one year of age; bnt no union had been effected. This result Sir Wm. Fergusson attributed to the omission to remove a projecting intermaxillary bone. He observed that the operation could not be said to have been really completed without the removal of the projection in all cases where it exists; because, as the teeth which it evolves never grow well, no good reason can be adduced for preserving it. The cleft was in the right side, and extended to the hard and soft palates.

Sir W. Fergusson operated on two other patients, adults, for cleft in the hard and soft palates. He directed his attention to the latter only, reserving the anterior portions of the fissure for a future occasion.

